# Supplementary material for: Morphology of ejecta features from the impact on asteroid Dimorphos
Source: Nat Commun. 2025 Feb 14;16:1601. doi: 10.1038/s41467-025-56551-0 (PMC11829021; doi:10.1038/s41467-025-56551-0)
Supplement: Supplementary file 1 — Supplementary Information [file 41467_2025_56551_MOESM1_ESM.pdf]

TITLE: Morphology of ejecta features from the impact on asteroid Dimorphos

SUPPLEMENTARY INFORMATION

Initial conditions for the simulation campaign (Supplementary Table 1 and Supplementary Fig. 1)

With reference to the dynamical simulations discussion in the Methods section, Supplementary Table 1 summarizes the velocity scaling power law (κ, γ) pairs selected to sample the random contribution v_{rand} of the particle initial velocity. A plot of the VSD is reported in Supplementary Fig. 1. The maximum velocity (corresponding to the minimum radius $R = 10^{-6}$ m) of the distribution is reported as well.

Supplementary Table 1: Velocity-size distribution parameters (κ,γ) pairs and maximum velocity for each dataset.

**Identifier**	**Coefficient** κ	**Exponent** γ	**Maximum velocity** v_{max} **[m/s]**
01	1.991e-2	0.4	5.00
02	9.953e-2	0.4	25.00
03	1.991e-1	0.4	50.00
04	9.953e-1	0.4	250.00
05	1.991e+0	0.4	500.00
06	1.991e-3	0.4	0.50
07	3.981e-3	0.4	1.00
08	9.953e-3	0.4	2.50
09	3.981e-2	0.4	10.00
10	3.981e-1	0.4	100.00
11	5.000e-6	1.0	5.00
12	2.500e-5	1.0	25.00
13	5.000e-5	1.0	50.00
14	2.500e-4	1.0	250.00
15	5.000e-4	1.0	500.00
16	5.000e-7	1.0	0.50
17	1.000e-6	1.0	1.00
18	2.500e-6	1.0	2.50
19	1.000e-5	1.0	10.00
20	1.000e-4	1.0	100.00
21	3.155e-3	0.7	50.00
22	6.310e-3	0.7	100.00
23	3.155e-5	0.7	0.50
24	6.310e-5	0.7	1.00
25	1.577e-4	0.7	2.50
26	3.155e-4	0.7	5.00
27	1.577e-3	0.7	25.00
28	6.310e-4	0.7	10.00
29	1.577e-2	0.7	250.00
30	3.155e-2	0.7	500.00

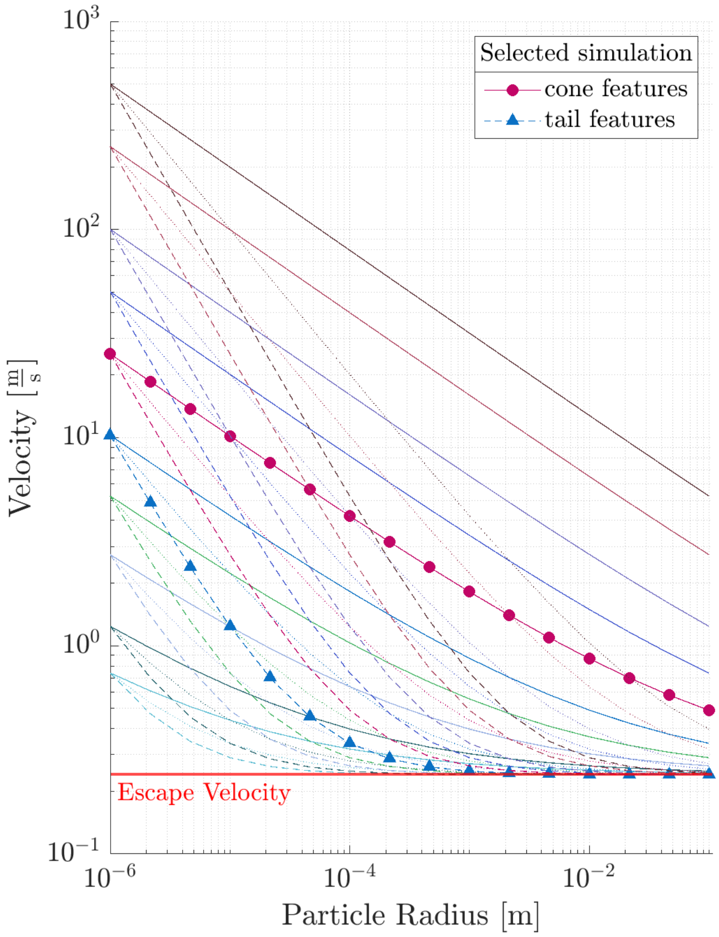

Supplementary Figure 1: **Velocity-size distribution curves**. Data is associated with parameters reported in Supplementary Table 1. The maximum velocity follows the velocity-size distribution in Eq. (2), with κ and γ given in Supplementary Table 1 The magenta solid line with the circular marker is the VSD associated with the cone-related features, whereas the blue dashed line with the triangular marker is the VSD related with the tail-related features. Note that the escape velocity is the velocity to escape from Didymos at the impact point. Selected simulation sets are IDs 19 (tail) and 02 (cone). Different colors indicate different initial condition sets. Source data are provided as a Source Data file.

Inhomogeneous structures in the ejecta cone (Supplementary Fig. 2)

Simulations of ejecta dynamics in this work consider homogeneous ejecta cone only. Figure 2 shows that synthetic images reproduce the general morphology and dynamical evolution of DART ejecta, apart from smaller-scale and irregular individual features, which would require a heterogeneous ejecta cone. As a representative example, we discuss here the case of the conspicuous feature “C1” (with reference to notation in ref. [1], and reported in Supplementary Fig. 2a), a seemingly rimmed hole in the DART impact ejecta cone, which can be traced to relatively great distance from the impact site[1]. Similar features were observed in experiments[2], where the holes through the ejecta cone were caused by boulders following low ejection trajectories passing through the more steeply ejected finer ejecta curtain (Supplementary Fig. 2b and 2c). Likewise, when the finer material of the curtain is impacted by the boulder a ring-shaped accumulation of material appears around the hole, similar to a crater rim. We propose the observed feature “C1” to be generated by the same mechanism, and basically independent of the difference in gravity. In the impact experiments at Earth gravity, after the boulder

has crossed the ejecta curtain, the hole moves outwards following the parabolic trajectories of the fine particles of the expanding ejecta curtain. After deposition, an irregularity is noted in the near field ejecta layer. However, after the DART impact (under low gravity conditions), the hole expands with the ejecta cone when it spreads out into space. In both cases, the formation is due to different trajectories of the fines in the ejecta curtain and of the much larger ejected boulders, which cross at a certain point. As the mechanism requires a strongly heterogeneous size distribution in the target material, the existence of holes through the ejecta curtain is itself a very useful indication of the target configuration. This may have applications to settings when only the ejecta cone is visible. Alternatively, the holes may be generated in a reverse situation with an expanding ejecta curtain striking an already orbiting boulder. We find this a less likely option for "C1" as the ejecta curtain would, in this case, leave a boulder behind when it expands, which is not observed.

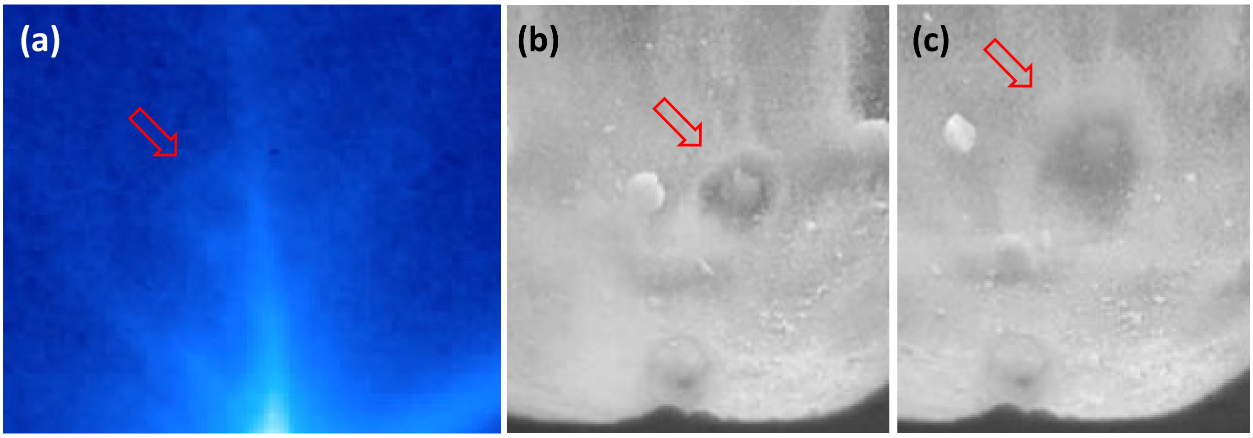

Supplementary Figure 2. Holes through the ejecta cone. (a) Circular feature "C1" (red arrow) observed after DART impact (adapted from Figure 3a in ref.[1]). (b and c) The hole formation (red arrows) in laboratory experiments by Ormö et al., (2022)(adapted from ref.[2] Suppl. Mtrl. 2). (b) The boulders pass the ejecta curtain on a lower trajectory leaving the hole behind. The boulder lands beyond the ejecta curtain. (c) The boulder disappears from the hole while the hole evolves radially in the plane of the ejecta curtain. A ring-shaped accumulation of material around the hole is generated (b) and expands (c) radially. The expansion will cause the hole to slowly fade. Adapted figures licence information: (a) https://creativecommons.org/licenses/by/4.0/ (b,c) https://creativecommons.org/licenses/by-nc-nd/4.0/

<u>Schematic overview of the methodology</u> (Supplementary Fig. 3)

A schematic overview of the methodology is reported in Supplementary Figure 3. Data and functional blocks are identified in the legend and detailed in the first paragraph of the Methods section.

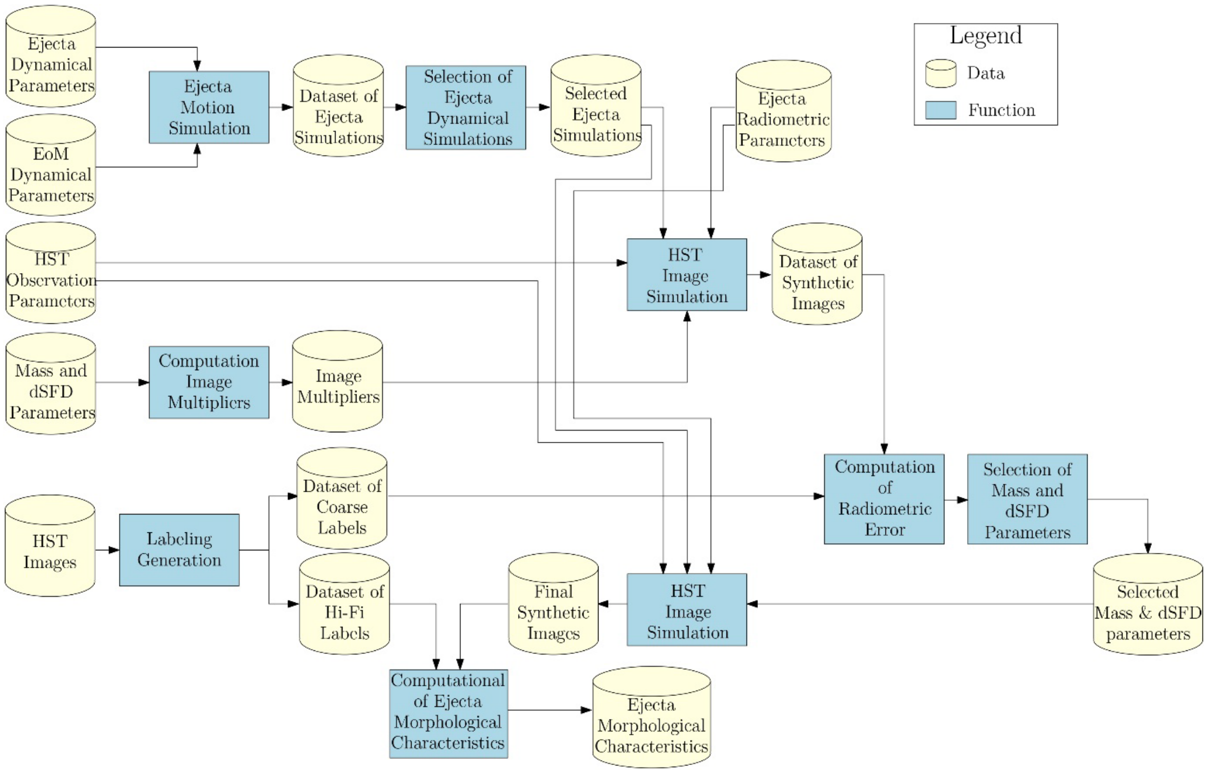

Supplementary Figure 3: **Schematic overview of the methodology.** Data (yellow) and Function (blue) blocks are highlighted, as well as their functional connection. Starting from the bottom left, HST images are labeled both with an high-fidelity and low-fidelity labelling. On the left upper part, the ejecta dynamics is simulated using the Equation of Motion (EoM) parameters and the Ejecta Dynamical parameters. This results in a dataset of ejecta dynamical simulations, among which the one reproducing the morphology of the ejecta feature is selected. This selected ejecta simulation is used, along with the ejecta radiometric parameters and the HST observation setting, to generate HST synthetic images. The latter are used to compute the radiometric error between the synthetic and real images in the areas identified by the low-fidelity labeling. The minimum of the radiometric error identifies the selected mass and dSFD parameters for both the spiral and the tail features. Finally, the selected mass and dSFD parameters are exploited to generated the final synthetic images, exploited to gather the morphological characteristics of the ejecta in the areas identified by the high-fidelity labelling.

REFERENCES

1. Li, J.-Y., Hirabayashi, M., Farnham, T.L. et al. Ejecta from the DART-produced active asteroid Dimorphos. Nature 616, 452–456 (2023). https://doi.org/10.1038/s41586-023-05811-4
2. Ormö, J., Raducan, S.D., Jutzi, M., Herreros, M.I., Luther, R., Collins, G.S., Wünnemann, K., Mora-Rueda, M., Hamann, C. Boulder exhumation and segregation by impacts on rubble-pile asteroids. Earth and Planetary Science Letters, Vol. 594 (2022). https://doi.org/10.1016/j.epsl.2022.117713
