# Supplementary material for: Morphology of ejecta features from the impact on asteroid Dimorphos
Source: Nat Commun. 2025 Feb 14;16:1601. doi: 10.1038/s41467-025-56551-0 (PMC11829021; doi:10.1038/s41467-025-56551-0)
Supplement: Supplementary file 2 — Description of Additional Supplementary Files [file 41467_2025_56551_MOESM2_ESM.pdf]

TITLE: Morphology of ejecta features from the impact on asteroid Dimorphos

Description of Additional Supplementary Files

File Name: Supplementary Video 1

Description: Ejecta evolution at HST scale, best-fit case (Figure 2, simulation set ID 02). Panels in Supplementary Video 1 report the evolution in time (from T0 to T0+14 days) of the ejecta features in HST images (top left), synthetic images from simulations (top right), feature identification (bottom left), HST images overlapped with synthetic images (bottom right).

File Name: Supplementary Video 2

Description: Ejecta evolution at HST scale, dedicated simulation to investigate the curvature in the tail (Figure 5h, simulation set ID 21). Panels in Supplementary Video 2 report the evolution in time (from T0 to T0+14 days) of the ejecta features in HST images (top left), synthetic images from simulations (top right), feature identification (bottom left), HST images overlapped with synthetic images (bottom right).

File Name: Supplementary Video 3

Description: Ejecta evolution close to Didymos system, 20 km field of view. Supplementary Video 3 shows the evolution of a subset of ejecta fragments at a scale closer to the system compared to HST scale, and within a field of view of 20 km (Figure 3, simulation set ID 02). The trajectory of ejecta simulation points is shown without any photometric adjustment. The mass of individual ejecta particles is reported, with reference to the colorbar attached.

File Name: Supplementary Video 4

Description: Ejecta evolution close to Didymos system, 100 km field of view. Supplementary Video 4 shows the evolution of a subset of ejecta fragments at a scale closer to the system compared to HST scale, and within a field of view of 100 km (simulation set ID 02). The trajectory of ejecta simulation points is shown without any photometric adjustment. The mass of individual ejecta particles is reported, with reference to the colorbar attached.

File Name: Supplementary Video 5

Description: Ejecta evolution close to Didymos system, 1000 km field of view. Supplementary Video 5 shows the evolution of a subset of ejecta fragments at a scale closer to the system compared to HST scale, and within a field of view of 1000 km (simulation set ID 02). The trajectory of ejecta simulation points is shown without any photometric adjustment. The mass of individual ejecta particles is reported, with reference to the colorbar attached.

File Name: Supplementary Video 6

Description: Ejecta evolution closer to Didymos system, 5000 km field of view. Supplementary Video 6 shows the evolution of a subset of ejecta fragments at a scale closer to the system compared to HST scale, and within a field of view of 5000 km (simulation set ID 02). The trajectory of ejecta simulation points is shown without any photometric adjustment. The mass of individual ejecta particles is reported, with reference to the colorbar attached.
